# Evaluation of Group B *Streptococcus* culture processing using a commercial flocked swab with and without automated sample processing instrument

**DOI:** 10.1128/spectrum.01249-25

**Published:** 2025-07-07

**Authors:** Richard D. Smith, Paul M. Luethy, J. Kristie Johnson

**Affiliations:** 1 Department of Pathology, School of Medicine, University of Maryland 12264, Baltimore, Maryland, USA; Indiana University School of Medicine, Indianapolis, Indiana, USA

**Keywords:** clinical microbiology, flocked swabs, automation, Group B *Streptococcus*

## Abstract

Group B *Streptococcus* (GBS) is the most common cause of neonatal sepsis. Screening for vaginal-rectal colonization of the mother is important to prevent exposure. The current standard diagnostic method for GBS is to collect a vaginal-rectal swab and inoculate in LIM broth to increase sensitivity. Use of flocked swabs such as Eswabs has become the recommended collection device due to their increased recovery rates and potential for automation. Despite being the recommended collection device, there is a lack of adequate literature providing guidance on processing of Eswabs for GBS cultures. This study sought to address this by investigating the optimal volume of transport media to inoculate in LIM broth, determining the limit of detection using the Copan WASP automated laboratory processor, and evaluating the accuracy of the WASP for recovering GBS using Eswabs. This study found that GBS can be recovered at multiple inoculation volumes except at 10 µL at a bacterial concentration of 10° CFU/mL, where it had a recovery rate of 80% (12/15). Moreover, when inoculating in LIM broth, the limit of detection for GBS using the WASP was 100% at 10^1^ CFU/mL. When using clinical specimens, WASP had 100% sensitivity and specificity. Overall, this study demonstrated that the WASP is an excellent automated method for processing GBS swabs. For laboratories without automation, this study demonstrates that any volume greater than 10 µL can recover GBS at a concentration greater than 10^0^ at 100% recovery rate compared to the gold standard of manual inoculation and plating.

**IMPORTANCE:**

Despite several published guidelines on how to process Group B *Streptococcus* (GBS), including type of swabs, media, incubation, and other molecular technology, there are no clear guidelines regarding the ideal volume of transport media to inoculate into LIM broth. Thus, this study investigated using manual and automated processing techniques. Moreover, as automation becomes a critical part of laboratory science, this study addresses and evaluates the use of automation for GBS.

## INTRODUCTION

*Streptococcus agalactiae*, also known as Group B *Streptococcus* (GBS), is the most common cause of neonatal sepsis and is acquired during vaginal birth; therefore, intrapartum screening for vaginal-rectal colonization of the mother is important to prevent exposure (1). Prevalence of colonization is estimated to be about 10–40% of women of birthing age, with 1–2% of babies being infected with GBS if the colonized mother is untreated (2). Fortunately, GBS is routinely susceptible to penicillin and other beta-lactam antibiotics (3). Thus, with routine testing, severe neonatal disease is easily preventable. The current standard diagnostic method for GBS is to collect a vaginal-rectal swab from pregnant women 35–37 weeks of gestation (4). Typically, GBS is identified in the clinical microbiology laboratory using selective broths such as LIM broth (Todd-Hewitt broth with colistin and nalidixic acid), where the broth is incubated for 24 h and then either subcultured to media (blood or CHROMagar) for GBS identification or used in NAAT (5).

A major pre-analytical aspect that impacts the recovery of GBS is the type of swab used for collection. Use of flocked swabs in combination with liquid-based transport medium such as an Eswab (Copan Diagnostics, Murrieta, CA) has been shown to significantly improve recovery of GBS compared to traditional fiber-wrapped swabs (6–8). Moreover, Eswabs allow for processing via an automated specimen processor or total laboratory automation, such as the COPAN WASP and BD Kiestra, which substantially reduces hands-on time for clinical microbiology staff processing these cultures (9). This is especially beneficial in overburdened or understaffed laboratories. Given the improved recovery of GBS and the ability to utilize laboratory automation, flocked swabs such as Eswabs have become the recommended swabs for GBS screening by the American Society of Microbiology (ASM) guidelines (5). The strengths of flocked swabs have been demonstrated in several brands of flocked swabs, but Eswabs have become a prominent brand. Despite published guidelines from ASM and other literature that thoroughly outline several approved methods for GBS processing, these guidelines fail to endorse the optimal volume of liquid transport medium to inoculate in LIM broth.

In comparison to traditional fiber-wrapped swabs where the entire swab is placed in LIM broth, there are limited data and guidelines on how to process Eswabs for GBS screening, leading to lack of standardization across multiple laboratories and, potentially, inaccurate results. Therefore, it is paramount to adequately determine the ideal processing methods for Eswabs. Overall, this study aims to address two major gaps in knowledge pertaining to GBS screening with Eswabs. First, this study investigates the ideal inoculation volume of transport media from Eswabs for GBS cultures, and second, it evaluates the performance of an automated processing method for GBS conducted by the Copan WASP (Copan Diagnostics, Murrieta, CA).

## MATERIALS AND METHODS

### Determination of optimal transport media volume to inoculate in LIM broth

To determine the ideal volume to inoculate into LIM broth, five different clinical isolates of GBS were collected and tested in triplicate. For each isolate and its replicate, a MacFarland of 0.5 (~10^8^ colony forming units per milliliter [CFU/mL]) of bacteria was created. Next, serial 1:10 dilutions were performed down to a concentration of 10° CFU/mL. Each dilution was subcultured to sheep blood agar (SBA) to confirm the concentration was accurate. For dilutions at the concentrations 10^0^, 10^1^, 10^2^, 10^3^, and 10^4^ CFU/mL, Eswabs were placed in dilution tubes for 1 min, then placed in transport media. Once in transport media, the swabs were thoroughly vortexed for 10 s. Several volumes of transport media were inoculated into 5 mL of LIM broth (Becton Dickinson, Sparks, MD): 1, 0.5, 0.25, 10 µL, and swab only (Table 1) for a total of 25 swabs. LIM broth was then incubated at 35°C for 24 h with 5% CO_2_. Subsequently, 10 µL of LIM broth was subcultured onto blood agar using a loop. Blood agar plates were then analyzed to see if GBS was recovered after 16–24 h of incubation.

**TABLE 1:** Performance of manual processing of Eswabs at different bacterial loads and transport media volumes

	1 mL	0.5 mL	0.25 mL	Swab only	10 µL
10^4^ CFU/mL	100% (15/15)	100% (15/15)	100% (15/15)	100% (15/15)	100% (15/15)
10^3^ CFU/mL	100% (15/15)	100% (15/15)	100% (15/15)	100% (15/15)	100% (15/15)
10^2^ CFU/mL	100% (15/15)	100% (15/15)	100% (15/15)	100% (15/15)	100% (15/15)
10^1^ CFU/mL	100% (15/15)	100% (15/15)	100% (15/15)	100% (15/15)	100% (15/15)
10° CFU/mL	100% (15/15)	100% (15/15)	100% (15/15)	100% (15/15)	**80%** (**12/15**)^*a*^

^*a*^ The bolded text indicates the volume and concentration where recovery was not 100%.

### Collection of clinical GBS Eswabs

Eswabs were collected prospectively from June 2024 to October 2024. Eswabs were processed for this study within 24 h of collection and were processed simultaneously by the clinical microbiology laboratory manually per standard operating procedure (SOP) and by the WASP. Specimens were collected for analysis under the University of Maryland, Baltimore Institutional Review Board (IRB HP-00109503). Collection included both specimens positive and negative for GBS.

### Processing GBS clinical cultures via Copan WASP

A preprogrammed method for processing GBS cultures was placed on the WASP. This method consisted of two different processes. The first process was the standard processing procedure with initial inoculation of LIM broth. Eswabs were placed on the WASP and aggressively vortexed. A 10 µL loop was used to inoculate a 2 mL Copan LIM broth tube. Another 10 µL of transport media was taken and directly plated to SBA without the use of LIM broth. The LIM broths were manually placed in the incubator at 35°C for 24 h with 5% CO_2_. The next day, the LIM broth was placed back on WASP, where 10 µL was plated to blood agar. The second automated method was a custom-built method programmed to directly plate GBS cultures to SBA without subculture to LIM broth. Despite direct plating not being endorsed by guidelines, since this method was already programmed, we included it in this study. To determine the limit of detection of the WASP to recover GBS, five different clinical isolates of GBS were collected and tested in triplicate at concentrations of 10^0^, 10^1^, 10^2^, 10^3^, and 10^4^ CFU/mL. Additionally, prospectively collected Eswabs from clinical specimens were processed manually by inoculating the entire transport media into LIM broth per our SOP and by the WASP to evaluate the recovery of GBS using WASP processing. Plates were read manually, and results were compared to culture results from the corresponding electronic medical records after processing according to our SOP. Our SOP includes manually inoculating the entire Eswab transport media into LIM broth, incubating for 24 h, and manually subculturing to SBA and manually reading for beta-hemolysis and formally identifying via MALDI-TOF MS.

### Statistical analysis

Sensitivity and specificity were calculated for direct plating and subculture after LIM broth compared to clinically reported results from manual plating per the laboratory SOP.

## RESULTS

### Determination of optimal transport media volume to inoculate in LIM broth

To identify the ideal volume of Eswabs transport media to inoculate in LIM broth, Eswabs were placed in solutions with concentrations of 10^0^, 10^1^, 10^2^, 10^3^, and 10^4^ CFU/mL of GBS. A total of five different clinical isolates from five different patients were used in triplicate. Tested volumes of transport media included 1 mL, 0.5 mL, 0.25 mL, 10 µL, and swab only. GBS was recovered from all volumes and concentrations except for 10° CFU/mL with 10 µL inoculated in LIM broth (Table 1). For this concentration, all three replicates from one isolate did not have GBS recovered. The presence of GBS was confirmed by plating the initial dilution and observing GBS growth on SBA.

### Evaluation of Copan WASP

First, to evaluate the performance of the WASP to recover GBS, the limit of detection was determined using contrived specimens with GBS. For subculture onto SBA without inoculation of LIM broth, GBS was recovered in 100% of Eswabs at concentrations of 10^2^ CFU/mL or greater. Only 80% (12/15) of isolates were recovered when the concentration of GBS inoculated into Amies transport media was 10^1^ CFU/mL and 20% (3/15) at 10° CFU/mL. The limit of detection for contrived isolates was a log lower for isolates subcultured into LIM broth, with 100% of isolates being recovered at initial concentrations of 10^1^ CFU/mL or greater and 80% (12/15) being recovered at 10° CFU/mL (Table 2).

**TABLE 2:** Limit of detection using the WASP automated plating system

	Growth on initial plate	Growth on LIM broth subculture
10^4^ CFU/mL	100% (15/15)	100% (15/15)
10^3^ CFU/mL	100% (15/15)	100% (15/15)
10^2^ CFU/mL	100% (15/15)	100% (15/15)
10^1^ CFU/mL	80% (12/15)	100% (15/15)
10° CFU/mL	20% (3/15)	80% (12/15)

After the limit of detection was determined, clinical isolates were prospectively collected to further evaluate the WASP recovery of GBS. A total of 76 clinical isolates were collected, including 63 GBS negative and 13 GBS positive as determined by standard manual processing. When using WASP to plate to SBA without inoculating to LIM broth, only 46.2% (6/13) of specimens positive for GBS by standard culture methods were recovered in culture. In contrast, when LIM broth was inoculated by WASP first, 100% (13/13) of clinical GBS-positive specimens recovered GBS in subculture, further highlighting the need for LIM broth. For all GBS-negative specimens, cultures processed by WASP with and without LIM broth inoculation were negative (63/63) (Table 3).

**TABLE 3:** Evaluation of processing via WASP using clinical specimens collected with Eswabs

	Growth on initial plate		Growth on LIM broth subculture	
	Positive	Negative	Positive	Negative
Positive by culture per current SOP (*n* = 13)	46.2% (6/13)	53.8% (7/13)	100% (13/13)	0% (0/13)
Negative by culture per current SOP (*n* = 63)	0% (63/63)	100% (63/63)	0% (63/63)	100% (63/63)

## DISCUSSION

This study is the first study to address several gaps in knowledge regarding the processing of commercial flocked swabs such as the Eswabs for GBS screening. The first gap this study addresses is the optimal volume of liquid transport media required to inoculate LIM broth and recover GBS. Despite several published studies and guidelines illustrating the importance of using flocked swabs, how to process these swabs has not been made clear (5). Eswabs contain Amies transport media that can absorb inhibitory substances and increase recovery of GBS to as high as a 100% recovery rate (10). In contrast, traditional fiber-wrapped swabs often trap microbes in the swab and do not contain transport media, which reduces the elution of the microbe and the recovery in culture. While the use of Amies transport media in flocked swabs has shown improved recovery, it is unclear what volume of this transport media must be added to LIM broth for ideal recovery. This study demonstrated that, when manually processing, 100% of GBS was recovered except at very low concentrations and when only 10 µL was inoculated in LIM broth. However, this failure to recover was seen in a single isolate for all three replicates, indicating a potential issue with the isolate or processing. Overall, based on the data from this study, we recommend inoculating 250 µL of transport media when manually processing GBS swabs. If automation is used, we recommend inoculating 10 µL into LIM broth.

Based on the results from manual processing, we hypothesized that the WASP may have a lower sensitivity for the recovery of GBS as it only inoculates 10 µL of Amies transport media into LIM broth. Moreover, since WASP also plates directly to SBA from the primary specimen, we expected these results to have even lower yields as LIM broth has shown to be an important enrichment media for GBS (11, 12). When conducting the limit of detection study, the results echoed our findings with manual processing and our expectations for processing without LIM broth subculture. When inoculating LIM broth on the WASP with 10 µL of Amies transport media, 100% recovery was observed for each concentration except 10° CFU/mL, where 80% (12/15) were recovered. While consistent with our findings of manual processing, our manual processing failed to recover one isolate in triplicate, while WASP failed to recover one replicate in three different isolates. As expected, the limit of detection of plating the swabs without inoculation to LIM broth first was a log higher of bacterial concentration. This further supported the need to utilize selective and enriched media for GBS, such as LIM broth, when utilizing automated processing of Eswabs.

While data with contrived specimens demonstrated that recovery of GBS diminishes at lower concentrations on the WASP, it is unclear if this would affect the processing of clinical specimens. The typical bacterial burden of GBS for colonized women is believed to be low; however, a formal determination of bacterial burden for those colonized is lacking (13–16). Furthermore, vaginal-rectal swabs have often been demonstrated to have high bacterial yields of GBS (13–16). With data lacking regarding the typical burden of GBS, testing with clinical isolates was critical. A total of 76 clinically collected GBS Eswabs, including 13 positives and 63 negatives, were used to evaluate WASP processing. No false positives were observed when samples were processed on the WASP. Additionally, using the WASP which inoculated 10 µL of transport media, 100% of GBS-positive specimens were recovered with LIM broth, but only 46.2% (6/13) were recovered without LIM broth enrichment. Although turnaround time is reduced by 24 h by removing the use of LIM broth, due to the low yield, processing on the WASP without enriched media it is not recommended based on this data. The data from processing clinical specimens were consistent with previously published data using the WASP; however, these studies used custom programming to inoculate 30 µL instead of 10 µL into LIM broth (9). Furthermore, Buchan et al. also directly plated to SBA, but a recovery rate of 87.5% was observed, which was much higher than in our study. This discrepancy may be attributed to the difference in clinical specimens or the higher volume of transport media plated.

This study had several strengths and weaknesses. A major strength of this study was the thorough investigation into processing methods for GBS screening cultures, something that has not been adequately addressed since the advent of flocked swabs such as Eswabs. This study included investigation into the volume of transport media needed to inoculate, the limit of detection studies, and the evaluation of an automated laboratory system. Despite the strengths of this study and the knowledge gap it addresses, there are some limitations. First, there is a limited sample size, particularly positive GBS specimens. The limited number of specimens was due to several specimens collected using non-flocked swabs. Despite the known advantages of flocked swabs, many clinics and other healthcare centers still use non-flocked swabs, perhaps due to familiarity or budgetary reasons. Furthermore, 17.1% of our specimens were positive, which is consistent with published colonization prevalence of 5–20% depending on region (17, 18). Another potential limitation is that isolates were collected only in spring and summer months; however, no seasonality of GBS colonization has been observed (19). The lack of positive samples is a major limitation of this study.

Overall, this study provides detailed insight regarding the processing of commercial flocked swabs for GBS screening. This study determines optimal volumes of transport media to inoculate in enriched and selective LIM broth, identifies a limit of detection for the automated Copan WASP that is compatible with Eswabs, and evaluates the recovery rate using the WASP with clinical specimens. Despite the knowledge this study provides, future research and studies to compare the use of the automated WASP system and other automated systems using LIM broth compared to other novel enrichment broths and molecular detection methods are necessary. These future analyses should not only compare the accuracy of these methods but also their impact on patient care and antibiotic use.
